# Folate-Functionalized DNA Origami for Targeted Delivery of Doxorubicin to Triple-Negative Breast Cancer

**DOI:** 10.3389/fchem.2021.721105

**Published:** 2021-08-16

**Authors:** Suchetan Pal, Tatini Rakshit

**Affiliations:** ^1^ Department of Chemistry, Indian Institute of Technology-Bhilai, Raipur, India; ^2^ Department of Chemical, Biological and Macromolecular Sciences, S. N. Bose National Centre for Basic Sciences, Kolkata, India

**Keywords:** DNA origami, folate receptor alpha, triple negative breast cancer, drug delivery, DNA nanotechnology

## Abstract

DNA origami has emerged as a versatile platform for diverse applications, namely, photonics, electronics, (bio) sensing, smart actuator, and drug delivery. In the last decade, DNA origami has been extensively pursued for efficient anticancer therapy. However, challenges remain to develop strategies that improve the targeting efficiency and drug delivery capability of the DNA origami nanostructures. In this direction, we developed folate-functionalized DNA origami that effectively targets and delivers doxorubicin (DOX), a well-known anticancer drug to the folate receptor alpha (FOLR1) expressing triple-negative breast cancer (TNBC) cells *in vitro*. We show that folate-functionalized DNA origami structure targets and kills FOLR1 overexpressing cells with better efficacy than nontargeted origami. We envision that this study will open up the possibility of target specific delivery of anticancer drug combinations using the versatile DNA origami nanostructures to the drug resistant cancer cells.

## Introduction

Although cancer is one of the deadliest diseases with a high mortality rate, it can potentially be cured if detected and treated at early stages. Once detected, most solid cancers require targeted therapy that selectively eradicate the cancer tissues while minimally affecting the normal tissues, this still remains a challenge. The field of “nanotheranostics” is emerging as an alternative to traditional methods for detecting and treating various types of cancer (Grodzinski et al., 2019). Nanotheranostic agents represent a union of imaging and therapeutic modalities in all-in-one formulation with a dimension of 10–500 nm. Due to the intrinsic nanoscale dimensions, these formulations selectively penetrate the leaky vasculature of cancer tissues. After the entry, nanoparticles are not efficiently cleared out due to impaired lymphatic drainage. This phenomenon is termed as Enhanced Permeability and Retention (EPR) effect or passive targeting (Maeda, 2012). Further surface modification of the nanoformulation with targeting agents such as small molecules, antibodies, peptides, and aptamers leads to further enhancement in selectivity to cancer cells or microenvironment, known as “active targeting” (Peer et al., 2007). Several advanced nanotheranostic formulations have been developed to detect and treat cancer in a single formulation (Xie et al., 2010). However, there is a constant need for developing nanotheranostic platforms made of biocompatible and biodegradable materials with minimal systemic toxicity for clinical translation (Rosenblum et al., 2018).

Deoxyribonucleic acid (DNA) is one of the fundamental molecules of nearly all life forms. Beyond biological relevance, the last 3 decades have seen an immense development in using DNA as construction material for building nanoscale materials, collectively referred to as “DNA nanotechnology” (Hong et al., 2017; Seeman and Sleiman, 2017). The advantages of using DNA as construction material are: 1) consistent and predictable structural parameters (2 nm in diameter, ∼50 nm of persistence length for double-stranded DNA); 2) highly conserved hydrogen bonding between nucleobases (A bonds with T and G bonds with C) that leads to totally predictable interaction and creation of branched DNA motifs; 3) inexpensive (bio) chemical synthesis. The advent of DNA origami technology, which uses folding of long viral “scaffold DNA strand” with chemically synthesized “staple DNA strands” into specific 2D and 3D shapes, have revolutionized the field of DNA nanotechnology. Due to these advantages, nanostructures with predetermined size, shape, and complexity are designed and fabricated in bulk quantity with relatively less time (Rothemund, 2006; Dietz et al., 2009; Douglas et al., 2009; Han et al., 2011). Due to inherent biocompatibility, ease of fabrication, and site-specific chemical modification, DNA origami structures have shown immense potential for various applications. Last few decades, DNA origami nanostructures were applied in nanofabrication, nanoplasmonics, nanoelectronics, catalysis, (bio) sensing, drug/gene delivery, and bioimaging applications (Dey et al., 2021). Excitingly, one of the significant applications has been in developing novel cancer theranostics, i. e., combination of cancer therapeutic and diagnostic agents (Nicolson et al., 2020). DNA origami embodies hundreds of staple strands in unique spatial locations. Using well-established conjugation chemistry, staple strands are modified to realize targeting, imaging, and therapeutic modalities (Madsen and Gothelf, 2019). DNA intercalating anticancer drugs such as Doxorubicin (DOX) selectively binds to DNA nanostructures, making DNA origami structures ideal nanocarriers for selective targeting and delivery of anticancer drugs (Zhao et al., 2012; Zhang et al., 2014; Zeng et al., 2018; Pan et al., 2020; Zeng et al., 2021).

**FIGURE 1:**
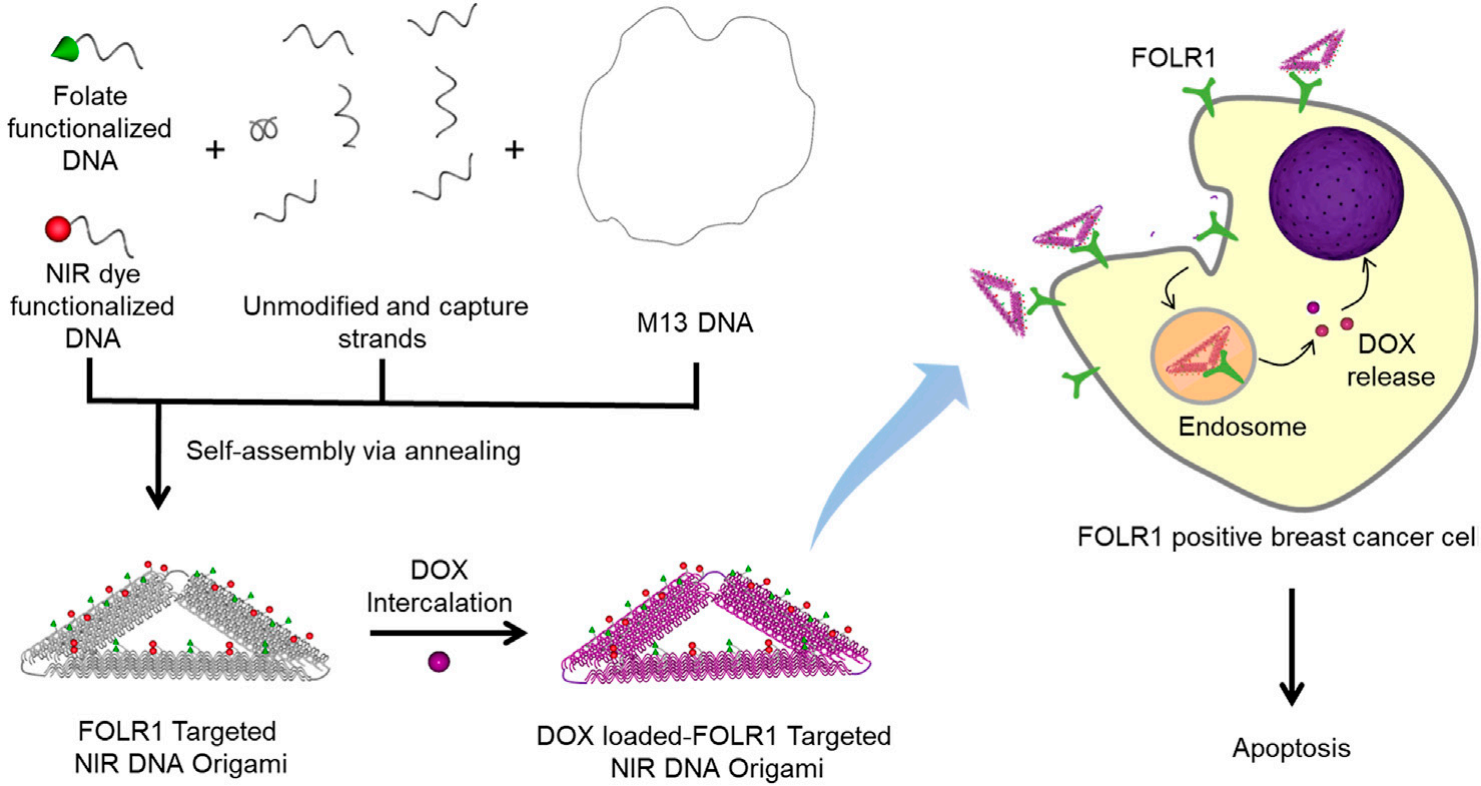
Schematic representation of folate functionalized DNA origami for targeted delivery of DOX. The FOLR1 targeted NIR DNA origami is fabricated by mixing M13 DNA with unmodified staple strands, captures strands, folate, and NIR dye functionalized DNA. Capture strands contain 15 base long single-stranded overhang in the 5′ end that is uniquely complementary to folate functionalized/NIR dye functionalized strands. A well-known anticancer drug, DOX, was intercalated in folate functionalized DNA origami structures. FOLR1 positive breast cancer cells selectively uptake folate functionalized DNA nanostructures via receptor-mediated endocytosis. DOX molecules are released from the endosomal compartment and translocate to the nucleus that result in cytotoxicity and cell death.

In this report, we demonstrate the selective targeting of FOLR1, a potential therapeutic target in different cancers with folate functionalized triangular DNA origami. FOLR1 is a membrane-protein which binds and transports folate, a vitamin essential for cell growth. To date, FOLR1 targeting has been investigated in ovarian and lung cancers (Toffoli et al., 1997; Boogerd et al., 2016). TNBC is the most aggressive form of breast cancer characterized by the lack of estrogen (ER), progesterone (PR) and human epidermal growth factor (HER2) receptors (Hartmann et al., 2007). Because of this, only a few targeted therapies of TNBC, for example, AKT and PARP inhibition, immunotherapy, Epidermal growth factor receptor (EGFR), Vascular endothelial growth factor receptor (VEGFR) are available till date (Kalimutho et al., 2015). Folate targeting is considered to be very promising for TNBC treatment (Necela et al., 2015). In this work, we explored selective site-specific modification of triangular DNA origami structures with folate molecules to target and deliver anticancer drug, DOX to TNBC cells for potential treatment (Figure 1).

## Materials and Methods

### Materials

All unmodified staple strands were purchased from Integrated DNA Technologies Inc. in 96-well plate format (final concentration of 200 μM/well) and mixed after arrival. All amino-functionalized DNA strands were also purchased from IDT DNA as HPLC purified grade. DyLight™ 690-B1-NHS were purchased from Thermofisher. Folic acid-PEG-NHS were purchased from Nanocs Inc. G-25 size-exclusion columns were purchased from GE Healthcare. Dimethylformamide (DMF), DOX hydrochloride (DOX⋅HCl) were purchased from Sigma aldrich. PE anti-FOLR1 (Folate Binding Protein) Antibody was purchased from Biolegend (Catalogue number 908304). 100 KDa molecular cutoff filter was purchased from Millipore. WST1 assay reagent was purchased from Merck Bioscience. Coster, black clear bottom 96-well plates were purchased from Sigma aldrich.

### Folate and NIR Dye Functionalization of Amine Modified DNA

Amine-modified DNA was functionalized with DyLight™ 690 (referred as NIR dye hereafter)/folate using well-established N-hydroxysuccinimide (NHS) ester chemistry (Supplementary Figure S1). In brief, 100 µL of 1 mM amine-modified DNA was added to 10 mM (100 µL) NHS ester of NIR dye/folate dissolved in anhydrous DMF and kept overnight at room temperature. Then, the reaction mixture was lyophilized and purified using G25 spin columns. The concentrations of purified labeled DNA were measured using a UV-Vis spectrophotometer and used for DNA origami fabrication.

### DNA Origami Design and Assembly

We used Rothemund’s original triangular origami design for this study(Rothemund, 2006). The structure has a total of 208 staple strands: 65 staple strands in each arm, 12 staple strands that connect arms and one staple strand for the loop. We selected 20 staple strands from each arm and extended the length with 15 nucleotide-long single-stranded overhang. Half of those 20 strands were complementary to the NIR dye, and folate functionalized DNA. Overall, we used 30 folate-capturing and 30 NIR dye-capturing staple strands, 148 unmodified staple strands, and NIR dye/folate functionalized strands to self-assemble folate targeted origami (detailed sequence information is provided in the SI). For the fabrication of nontargeted DNA origami, amine functionalized DNA strands were used in the place of folate functionalized strands. To assemble DNA origami, single-stranded M13mp18 DNA (New England Biolabs, 7,249 nt length) was mixed with normal and NIR dye/folate-capturing staple strands, NIR dye/folate functionalized strands in 1:10 ratio in 0.5 × TAE-Mg^2+^(20 mM Tris base pH 8.0, 10 mM acetic acid, 1 mM EDTA, and 6.25 mM magnesium acetate) buffer. The resulting solution was annealed from 95 to 4°C for the correct folding of DNA origami structure. The structures were subsequently purified three times by microcon centrifugal filtration devices (100 kD MWCO filters, Millipore) to remove the excess single-stranded DNA.

### DNA Origami Characterization

Agarose gel electrophoresis was performed in 1.5 % agarose gel (stained with ethidium bromide) at room temperature, and the images were collected using a gel imaging setup (Bio Rad, ChemiDoc). AFM imaging of DNA origami was performed in tapping mode under buffer. 30 μL of 1 mM DNA origami sample in 0.5 × TAE-Mg^2+^ (20 mM Tris base pH 8.0, 10 mM acetic acid, 1 mM EDTA, and 6.25 mM magnesium acetate) buffer was deposited on a freshly cleaved mica substrate and left to adsorb for 10 min. The sample was washed three times with filtered 1 × TAE- Mg^2+^ (40 mM Tris base pH 8.0, 20 mM acetic acid, 2 mM EDTA, and 12.5 mM magnesium acetate) buffer. The samples were imaged with tapping mode AFM (Asylum Research MFP-3D-BIO, Oxford Instruments) under 10 mM PBS with Olympus BL-AC40TS AFM probe (resonance frequency, 25 kHz under buffer; spring constant, 0.09 N m^−1^). The height value was obtained from the difference between the peak point in the cross-section and the average baseline.

### Cell Culture

Human breast cancer cells namely, MDA-MB-468 and MDA-MB-231 were obtained from National Centre for Cell Science (NCCS), Pune, India, and cultured in folate-free Leibovitz-15 media supplemented with 10% fetal bovine serum (FBS), 2 mM l-glutamine, 1% penicillin and streptomycin in a humidified atmosphere of 5% CO_2_ at 37°C. After seeding overnight in cell culture plates, desired concentrations of folate targeted, nontargeted, and DOX loaded DNA origami were added with fresh culture media and further incubated for different time durations. Post incubation, cells were washed three times with PBS and harvested for flow cytometry or fixed for confocal fluorescence imaging.

### Flow Cytometry

Flow cytometry-based DNA origami uptake studies were carried out by seeding 1 × 10^5^ cells in 6-well plates and waiting for 24 h to reach 70–80% confluence. The growth medium was replaced with a new medium containing desired concentration of labeled DNA origami. After the incubation for specific time duration, cells were detached with 0.25% trypsin and 0.05% EDTA in 10 mM PBS. The cells were washed twice with 10 mM PBS before it was measured using a flow cytometer (BD LSR II flow cytometer, BD Biosciences). All flow cytometry data were analyzed by FlowJo software.

### Cell Uptake Studies by Confocal Microscopy

Confocal microscopy of the fixed cells was done in a Leica-SP8 confocal fluorescence microscope. Cells were fixed in 4% paraformaldehyde in 10 mM PBS and stained with Hoechst® 33342 (1 μg/ml, for nucleus) and wheat germ agglutinin AF488/633 (5 μg/ml, for cell membrane) before imaging. Images were processed and quantified using Imaris 9.1.2 (Bitplane) software.

### DOX Loading and Cytotoxicity Studies

A DOX solution (1 µM in 10 mM PBS) was incubated with 10 nM folate functionalized DNA origami structures overnight. The resulting solution was centrifuged at 6000 rpm at RT for 30 min. The dark red pallet in the centrifuge tube was collected and the supernatant was used to determine the unbound DOX concentration by measuring DOX at 480 nm with a microplate reader (SpectraMax i3x Multi-Mode Microplate Reader, Molecular Devices). DOX loading efficiency was calculated using the formula below:

DOX loading=Initial [DOX]−final [DOX]in supernantant

$$\mathrm{DOX loading efficiency}=\;\frac{\mathrm{DOX loading}}{\left[\mathrm{Origami}\right]\;}$$

The cytotoxicity of DOX-loaded folate-targeted origami was evaluated for MDA-MB-468 cells seeded in 96 well plates using WST1 reagent. The cells were incubated with DOX-loaded folate functionalized DNA origami structures or free DOX for 24 h. After incubation, cells were gently washed, and WST1 reagent was introduced according to the manufacturer’s specification. The absorbance was measured at 440 nm using a microplate reader. Cell viability was calculated by the following equation:

$$Cell\;viability\left(\%\right)=\frac{A_{DOX}-A_{blank}}{A_{control}-A_{blank}}\times 100$$

Where A_DOX_ was absorbance of DOX treated cells, A_control_ was the absorbance of the cells incubated with the culture medium only, and A_blank_ was the absorbance of the culture medium. DMSO treated cells were used as positive control.

## Results and Discussion

### Fabrication and Characterization of Folate-Functionalized DNA Origami

**FIGURE 2:**
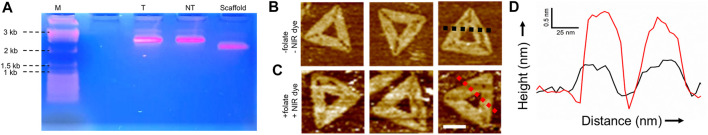
Characterizations of trianglular DNA origami nanostructures: **(A)** Agarose gel electrophoresis of M13 scaffold strand (scaffold), targeted DNA origami (T), and nontargeted DNA origami (NT) showing the correct folding of DNA nanostructures. **(B, C)** AFM topography images of DNA origami structures without and with folate and NIR dye modified strands, respectively. Scale bar:50 nm. **(D)** Height profiles of black trace in figure **(B)** and red trace in figure **(C)** show ∼1 nm height difference due to presence of folate, and NIR dye functionalized strands.

We assembled triangular origami structures by mixing M13mp18 DNA with 60 modified (capture strand) and 148 normal staple strands. We added a 15 nucleotide long single-stranded extension to 60 modified staple strands that were uniquely complementary to folate and NIR dye functionalized strands (see SI for details). The formation of the DNA origami was verified with agarose gel electrophoresis, as shown in Figure 2A. Both the FOLR1targeted (lane T) and nontargeted (lane NT) DNA origami ran slower than the M13 scaffold strand (lane scaffold), suggesting correct folding of the structure. We also imaged DNA origami structures assembled with and without folate and NIR dye functionalized strands in an Atomic Force Microscope (AFM), as shown in Figures 2B,C. The height profile of the folate and NIR dye functionalized strands functionalized DNA origami was found to be having ∼1 nm greater height than the origami without such modification, as depicted in Figure 2D. This was due to dangling of the 15-nucleotide long modified double strands that increased the thickness of the DNA origami. We further carried out agarose gel electrophoresis of DNA origami with and without capture strands and imaged under UV-light and NIR excitation. We found that in the presence of capture strands, NIR dye functionalized strands were incorporated in the structure (Supplementary Figure S2). These results unequivocally revealed that folate and dye modifications were indeed successful.

### Cellular Targeting of Folate-Functionalized DNA Origami

**FIGURE 3:**
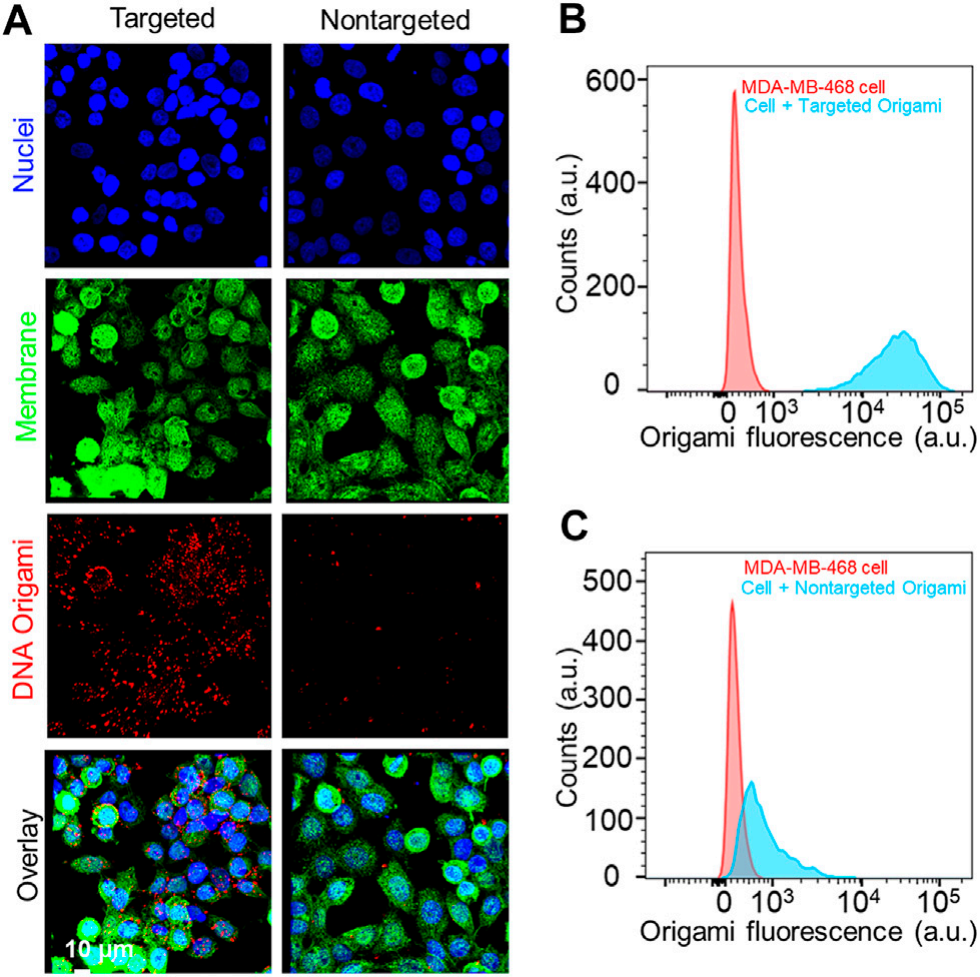
**(A)** Fluorescence microscope images of MDA-MB-468 cells incubated with 10 pM of targeted and nontargeted DNA origami structures for12 h. The red signal from DNA origami clearly showed a higher uptake of the targeted origami. Cell nucleus and membrane was stained with Hoechst® 33342 (blue) and wheat germ agglutinin, AF488 conjugate (green), respectively. **(B, C)** Flow cytometry-based analysis also reflected the same trend, enhanced uptake of targeted origami compared to nontargeted origami to the MDA-MB-468 cells.

After the successful fabrication of folate functionalized DNA origami, we sought to target TNBC cells. There exists a significant variation in FOLR1 expression level in breast cancer cell lines. Recently, expression of FOLR1 at mRNA level was measured in well-established TNBC cell lines, highlighting the diversity in the expression levels (Necela et al., 2015). We selected MDA-MB-468 and MDA-MB-231 cells to understand the FOLR1 expression levels. We carried out flow cytometry and fluorescence imaging-based measurements to determine the FOLR1 expression levels in the TNBC cells using a PE-labeled FOLR1 primary antibody (Supplementary Figure S3). These results revealed that the expression of FOLR1 in MDA-MB-231 cells was relatively low, which is consistent with previous observations(Necela et al., 2015). Therefore, for this study, we chose MDA-MB-468 to demonstrate FOLR1 targeting. To determine the targeting capability of DNA origami structures, we incubated MDA-MB-468 cells with 10 pM targeted and nontargeted DNA origami for 12 h at 37^o^C. Post incubation, we imaged the cells in a confocal fluorescence microscope. We found that intracellular fluorescence intensity was significantly higher for targeted DNA origami than nontargeted DNA origami (Figure 3A). We also carried out flow cytometry of the cells after 12 h incubation. We observed ∼40 times higher intracellular fluorescence in targeted DNA origami compared to nontargeted DNA origami, (Figures 3B,C). These results clearly suggested that the uptake was indeed facilitated by targeting of FOLR1 receptors.

### Cellular Uptake and Cytotoxicity of Folate-Functionalized DNA Origami

**FIGURE 4:**
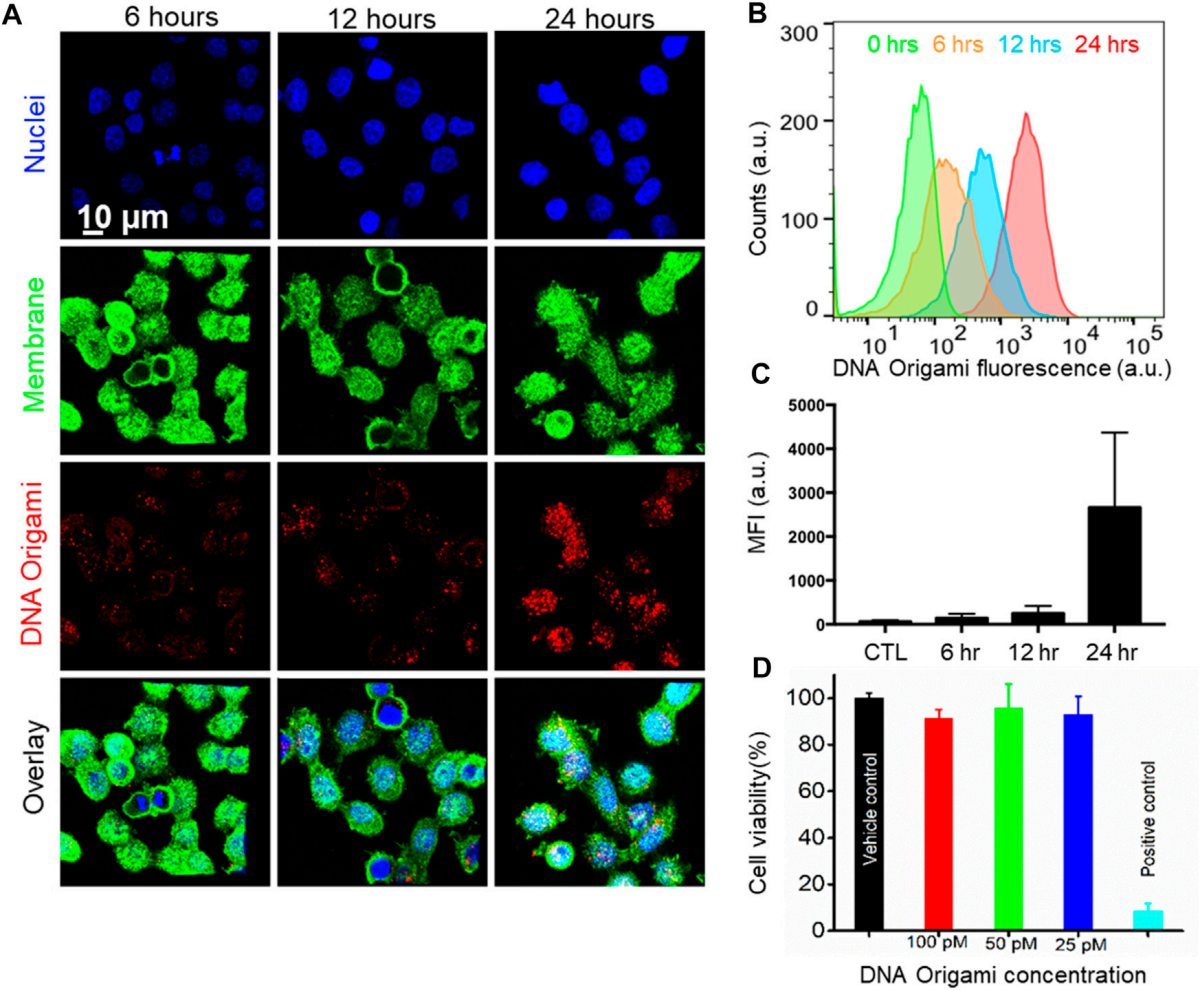
**(A)** Confocal Fluorescence microscope images of time-dependent targeted DNA origami uptake. MDA-MB-468 cells were incubated with 10  pM of targeted DNA origami structures for 6, 12, and 24 h respectively, suggesting a higher cellular uptake with increasing time; Cell nucleus and membrane were stained with Hoechst® 33342 (blue) and wheat germ agglutinin, AF488 conjugate (green), respectively. **(B, C)** Flow cytometry-based measurement also showed enhanced intracellular fluorescence with increasing time. **(D)** WST1 Cell viability assay validated no significant toxicity up to 100 pM targeted DNA origami.

**FIGURE 5:**
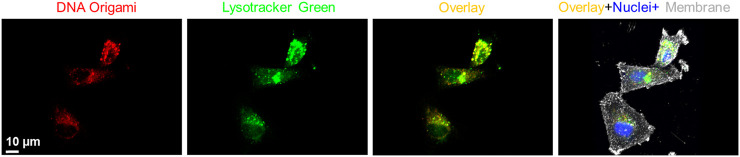
Fluorescence co-localization of DNA origami fluorescence (red) and a well-known lysosome marker, lysotracker green, showed significant localization of DNA nanostructures in the lysosomal compartments. Cell nucleus and membrane were stained with Hoechst® 33342 (blue) and wheat germ agglutinin, AF633 conjugate (grey), respectively.

Next, we investigated time-dependent uptake of the folate-functionalized DNA origami structures. We incubated the MDA-MB-468 cells with 10 pM targeted DNA origami structures for 6, 12, and 24 h. We carried out confocal fluorescence imaging to quantify the intracellular DNA origami concentrations, shown in Figure 4A. The cell membrane and nuclei were visible in green and blue fluorescence, respectively. The intracellular red fluorescence due to DNA origami gradually increased with time. To quantify this observation, flow cytometry based measurements were performed (Figure 4B,C). The fluorescence intensity increased over time and drastically enhanced between 12 and 24 h, as verified by the confocal fluorescence measurements. We further carried out WST1 assay to determine the cytotoxicity of the targeted DNA origami on the cell viability. We incubated the MDA-MB-468 cells with various concentrations of targeted DNA origami (25, 50 and 100 pM) and incubated the cells for 24 h. The cytotoxicity data clearly demonstrated the viability was not compromised due to the DNA origami intrusion, (Figure 4D). We further verified the cellular localization of the origami nanostructures as at 12 h time point, the fluorescence was localized in the puncta, as shown in Figure 5. This is well-established that DNA nanostructures enter endosomal sacks after the uptake, and over time, endosomes mature to lysosomes (Wang et al., 2018). Therefore, we used a well-known lysosome marker lysotracker green DND-26 to determine if the DNA origami nanostructures were localized in lysosomes. Fluorescence images revealed red fluorescence for DNA origami were indeed coincided with green fluorescence of lysotracker green suggesting a majority of DNA origami ended up going in low pH lysosomal compartments.

### Targeted Delivery of DOX to TNBC Cells

**FIGURE 6:**
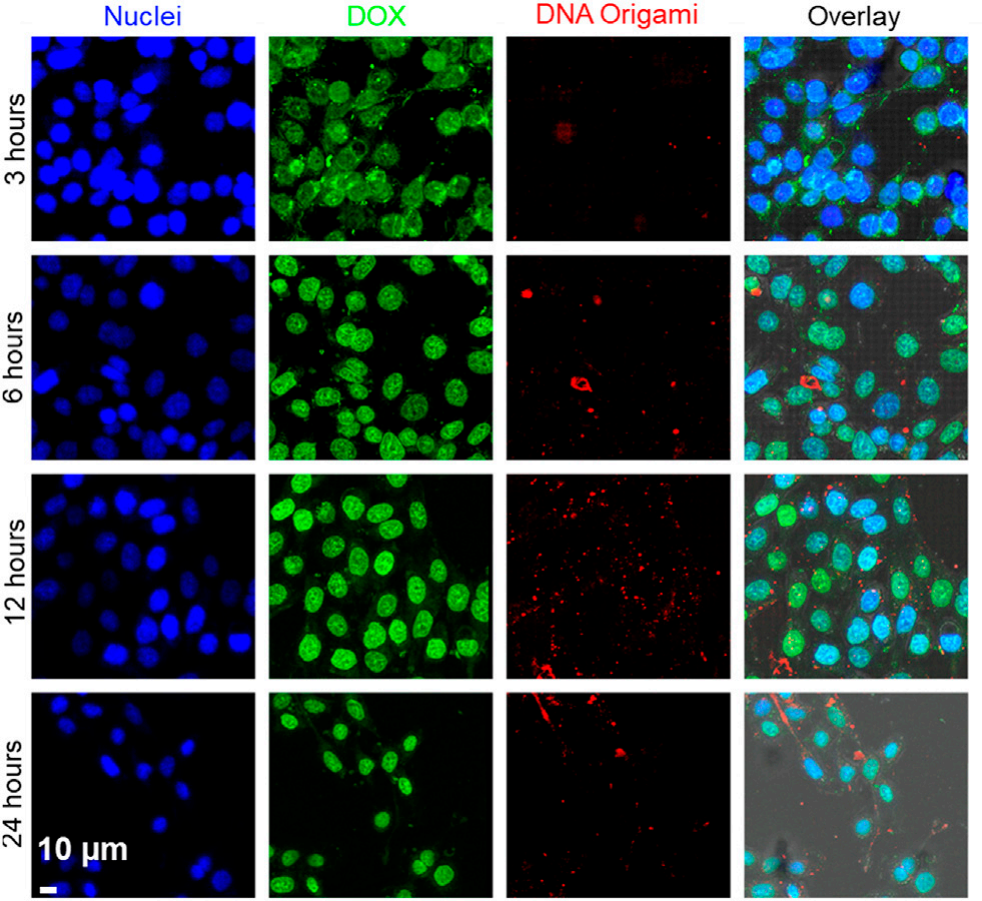
Time-dependent delivery of DOX to the MDA-MB-468 cells. DOX fluorescence is shown as green, and DNA origami fluorescence is shown as red. Images showed that at 3 h, DOX was localized in the cytoplasm. With increasing time (6–24 h), DOX was released from DNA origami and translocated to the nuclei. Cell nuclei was stained with Hoechst® 33342 (blue). Scale bar: 10 µm.

Inspired by the targeted delivery of DNA origami to MDA-MB-468 cells, we incorporated a well-known anticancer drug DOX. The use of DOX is limited by severe toxicity on noncancerous cells, such as cardiotoxicity and neurotoxicity (Tacar et al., 2013). Therefore, specific delivery of DOX to cancer cells has been improved by several nanoformulation such as silica nanoparticles, polymeric nanoparticles, liposomes, protein, DNA-nanostructures (Kanapathipillai et al., 2014). Recently, folate-targeted DNA nanocage was used to deliver DOX to FOLR1 positive cancer cells (Raniolo et al., 2018). These encouraging findings led us to use folate-functionalized DNA origami for DOX delivery applications to TNBC cells. First, we loaded DNA nanostructures with DOX by incubating 1 µM DOX solution with 10 nM DNA origami solution at RT. Unbound DOX was removed using centrifugation. We estimated that, there were ∼300 molecules of DOX noncovalently attached to each DNA origami as described in the Methods section. The DOX incorporation slightly changed the shape of DNA origami, as shown in Supplementary Figure S4, due to changes in the helical twist in DNA origami structures owing to DOX incorporation. We also carried out native agarose gel electrophoresis to verify the binding of DOX with DNA origami structures (Supplementary Figure S5). We incubated MDA-MB-468 cells with the DOX-loaded FOLR1-targeted DNA origami structures for various time intervals (3, 6, 12, 24 h). We imaged the cells under a confocal fluorescence microscope (Figure 6). We observed the fluorescence signal (green) corresponding to DOX was present at 3 h time point primarily in the cytoplasm. However, at 6 h onwards, DOX molecules were more localized in the nuclei. DNA origami signal (red) always remained in the cytoplasm, presumably in lysosome. Therefore, DOX molecules leached from the DNA origami nanocarriers and escaped the endosomal compartment. Due to the high affinity of DOX with double-stranded DNA, DOX molecules specifically localized to the nucleus (Zeng et al., 2018). The imaging results suggested, DOX was released from the DNA origami structures in the endosome and escapes endosomal compartment to translocate to the nuclei which is consistent with previous findings (Jiang et al., 2012; Zhao et al., 2012; Zeng et al., 2018).

### Cytotoxicity of DOX-Loaded Folate-Targeted DNA Origami to TNBC Cells

**FIGURE 7:**
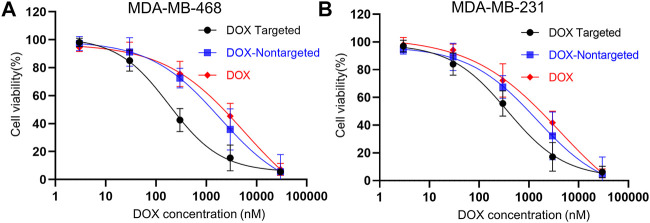
**(A)** Dose-dependent cytotoxicity of MDA-MB-468 cells and **(B)** MDA-MB-231 cells incubated with DOX-loaded targeted origami (black), DOX-loaded nontargeted origami (blue), free DOX (red) for 24 h. All the plots showed a sigmoidal decrease in viability with increasing DOX concentrations. For MDA-MB-468 cells, the IC50 values for DOX loaded targeted origami, DOX loaded nontargeted origami and free DOX were 182 nM, 1861 and 5740 nM, respectively. For MDA-MB-231 cells, the IC50 values for DOX loaded targeted origami, DOX loaded nontargeted origami and free DOX were 371 nM, 1440 nM and 4188 nM, respectively This implied higher efficacy of folate-targeted DOX delivery was achieved with DOX-loaded folate-functionalized DNA origami nanostructures.

Further, we investigated DOX dose-dependent cell viability using WST1 cytotoxicity assay. We incubated MDA-MB-468 and MDA-MB-231 cells with increasing concentrations of DOX, DOX-loaded folate targeted, and nontargeted DNA origami. After 24 h of incubation, we found that with increasing concentration of DOX, cell viability decreased following a sigmoidal pattern in all three DOX formulations (Figures 7A,B). For FOLR1 overexpressing MDA-MB-468 cells, the measured IC50 value for only DOX-loaded folate functionalized formulation was found to be ∼31 and ∼10.2 times lower than free DOX and nontargeted DNA origami, respectively. Further, for FOLR1 low expressing MDA-MB-231 cells, the IC50 values of DOX loaded folate functionalized DNA origami was found to be ∼11.3 and ∼3.9 fold lower than that of DOX loaded nontargeted DNA origami and free DOX respectively. The IC50 values of the free DOX and DOX-loaded nontargeted formulations were comparable to previous report (Zhao et al., 2012). These results implied that folate-functionalized DNA origami delivered DOX more efficiently with higher specificity to the folate overexpressing TNBC cells compared to the free DOX due to its enhanced uptake.

## Conclusion

We demonstrated the functionalization of DNA origami with folate molecules which has great potential to target FOLR1 positive TNBC cells with better efficiency and higher specificity compared to the nontargeted DNA origami structures. Our data revealed that, the uptake of the folate-functionalized DNA origami did not cause any severe cytotoxicity to the cells. Additionally, the dose of the drug (DOX) required to kill the cancer cells was ∼31 fold lower when folate-functionalized DNA origami mediated delivery was employed. This could be linked to the inherent affinity of DOX molecules to bind the DNA origami structures to make it more soluble. Collectively these results demonstrated the superiority of folate-functionalized DNA origami as a theranostics agent *in vitro.* For future aspects, the modularity of DNA-based design may aid in attaching suitable aptamer and antibody molecules for more specific targeting and delivery, although the long-term systemic toxicity of these constructs should be thoroughly tested.

## Data Availability

The original contributions presented in the study are included in the article/Supplementary Material, further inquiries can be directed to the corresponding author.
