# Supplementary material for: Folate-Functionalized DNA Origami for Targeted Delivery of Doxorubicin to Triple-Negative Breast Cancer
Source: Front Chem. 2021 Aug 16;9:721105. doi: 10.3389/fchem.2021.721105 (PMC8415400; doi:10.3389/fchem.2021.721105)
Supplement: Supplementary file 1 [file DataSheet1.DOCX]

Supplementary Material

**Folate Functionalized DNA Origami for Targeted Delivery of Doxorubicin to Triple-negative Breast Cancer**

**Suchetan Pal^1*^, Tatini Rakshit^2^**

^1^ Department of Chemistry, Indian Institute of Technology-Bhilai, Raipur, India

^2^ Department of Chemical, Biological & Macromolecular Sciences, S. N. Bose National Centre for Basic Sciences, Kolkata 700106, India

*** Correspondence:** suchetanp@iitbhilai.ac.in

## Supplementary Figures


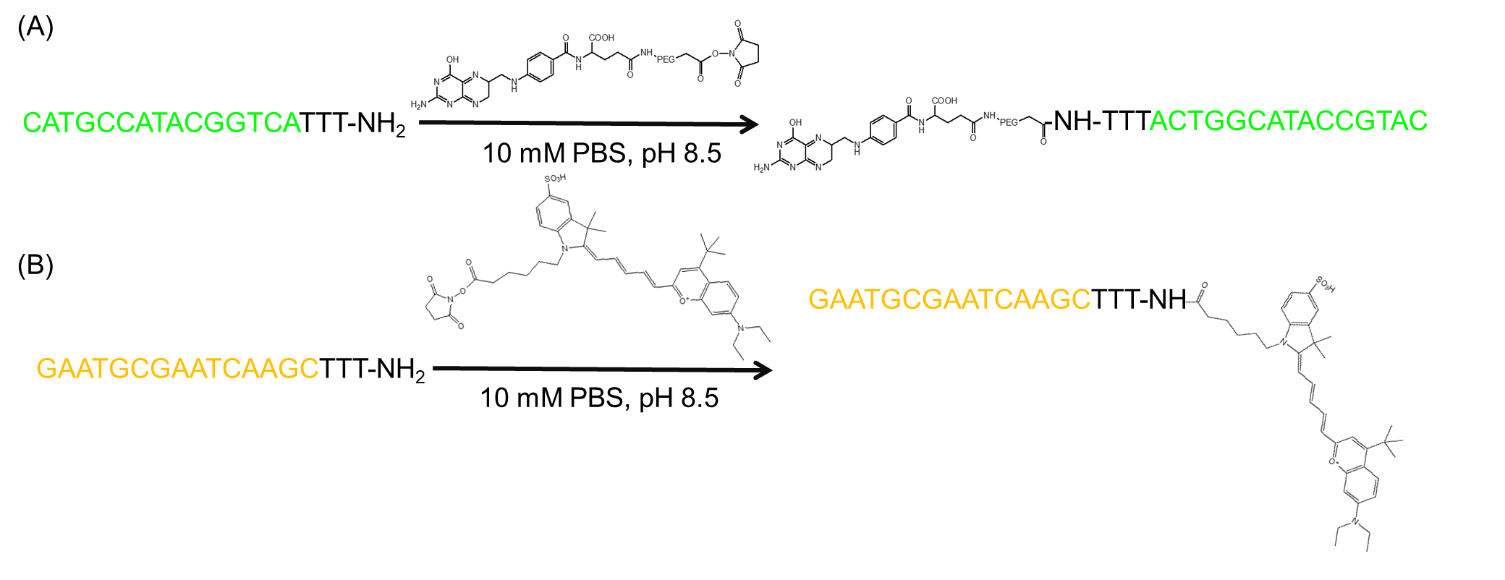


**Supplementary Figure S1.** Amine reactive NHS chemistry used for this study. (A) NHS ester of folate was reacted with the amine terminated strand (B) NHS ester of DyLight™ 690-B1 reacted with an amine terminated strand in 10 mM PBS (pH 8.5) at room temperature. The modified strands were purified using G25 column.


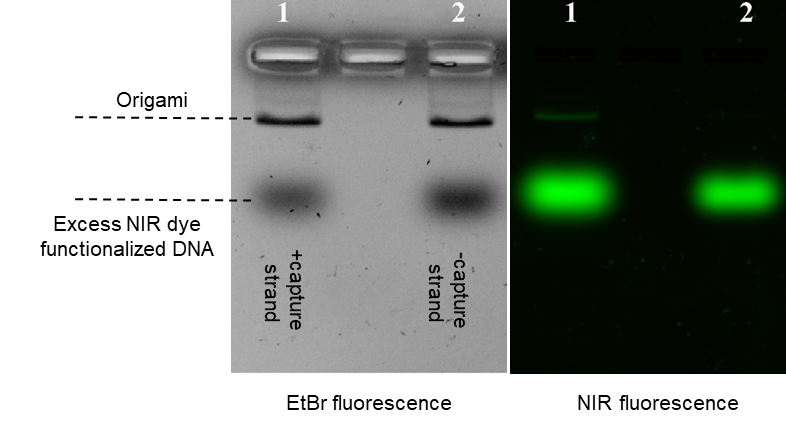


**Supplementary Figure S2.** Native 1.5 % agarose gel electrophoresis of DNA origami with (lane 1) or without (lane 2) NIR dye capture strands. The UV-visible scan of the gel shows formation of DNA origami structure. NIR fluorescence scan show that NIR dye-functionalized strands bind to the DNA origami displaying capture strands.


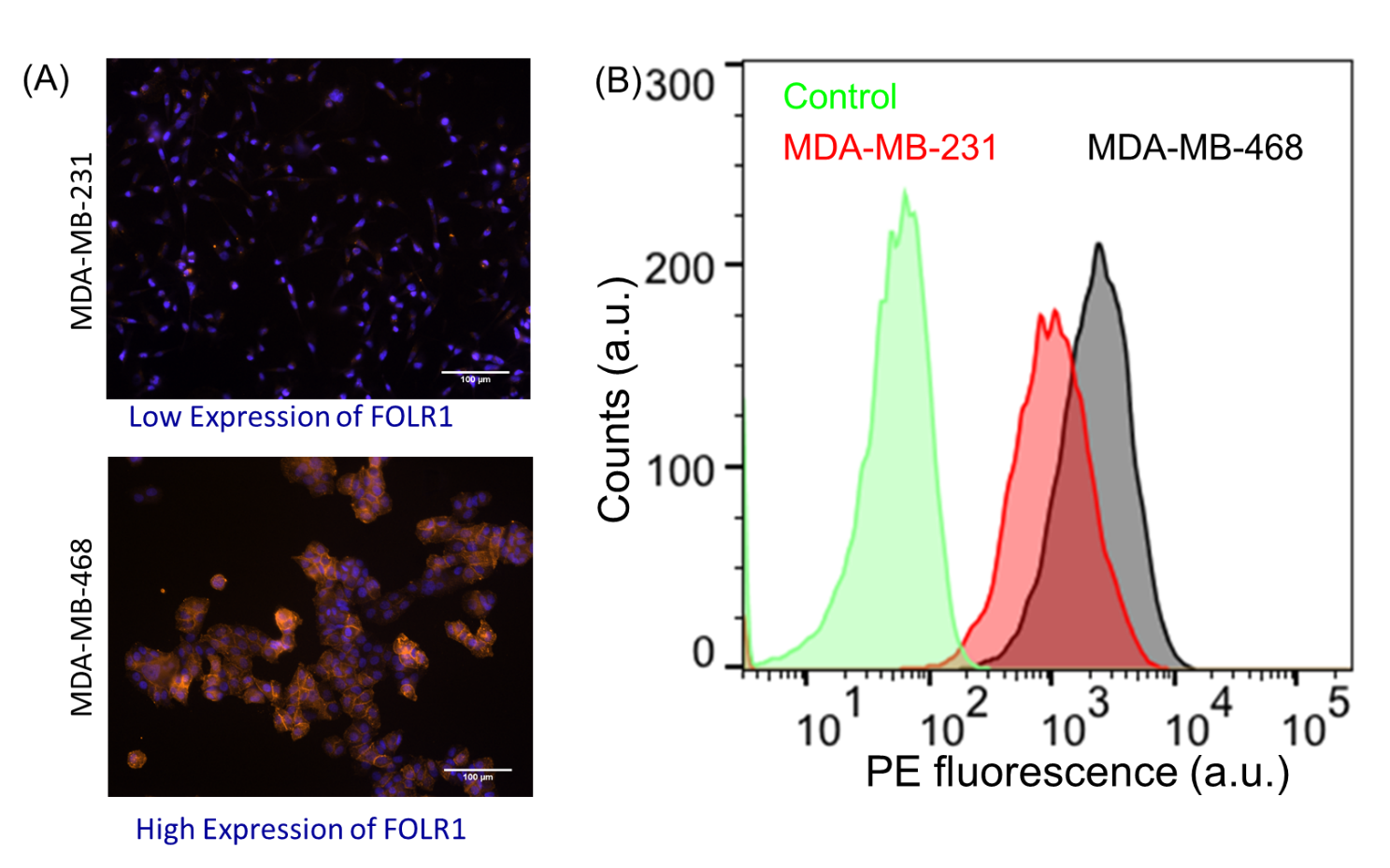


**Supplementary Figure S3.** Assessment of expression of FOLR1 using a PE labeled FOLR1 antibody. (A) Fluorescence images show higher expression of FOLR1 in MDA-MB-468 compared to MDA-MB-231 cells. (B) Flow cytometry-based measurements also show an increase FOLR1 expression in MDA-MB-468. These results collectively show the inherent variability of FOLR1 expression in TNBC cells.


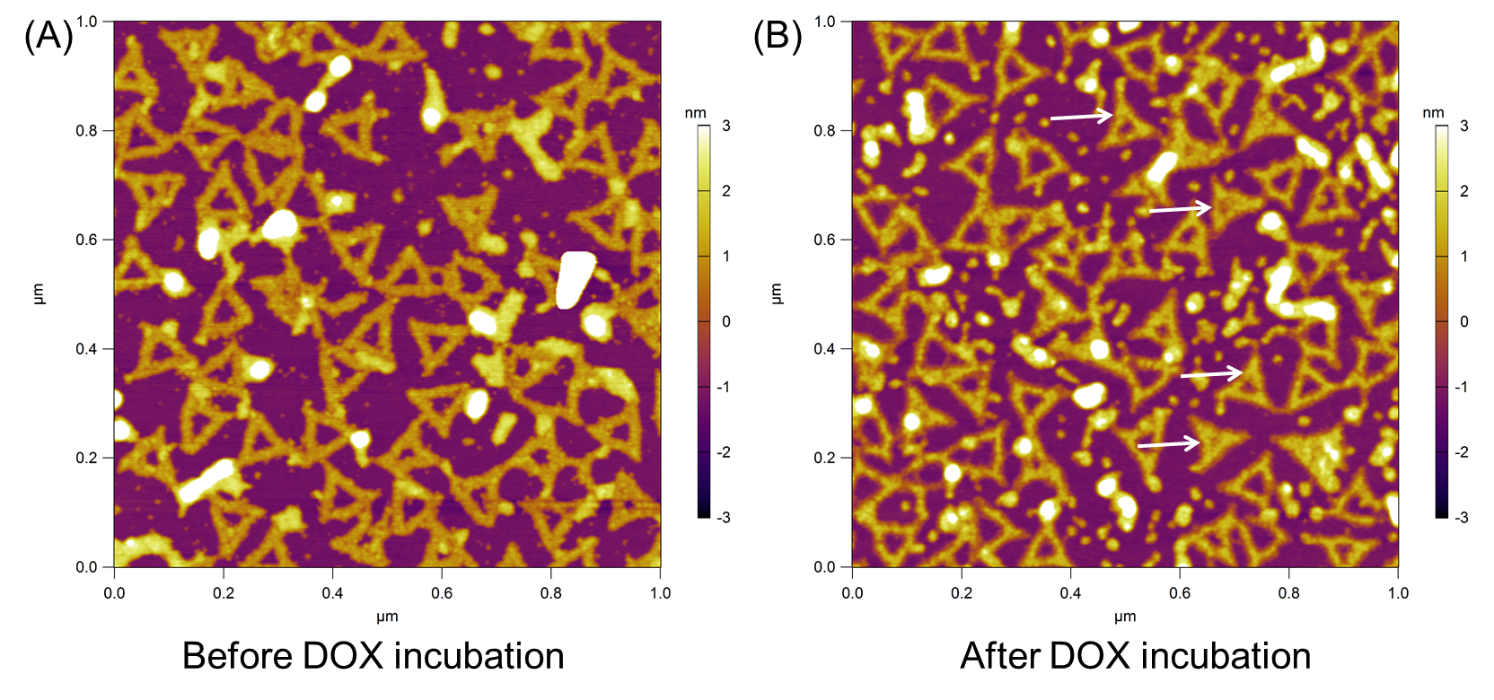


**Supplementary Figure S4.** AFM images of DNA origami (A) before DOX incubation, and (B) after DOX incubation showing structural deformation (white arrow).

**
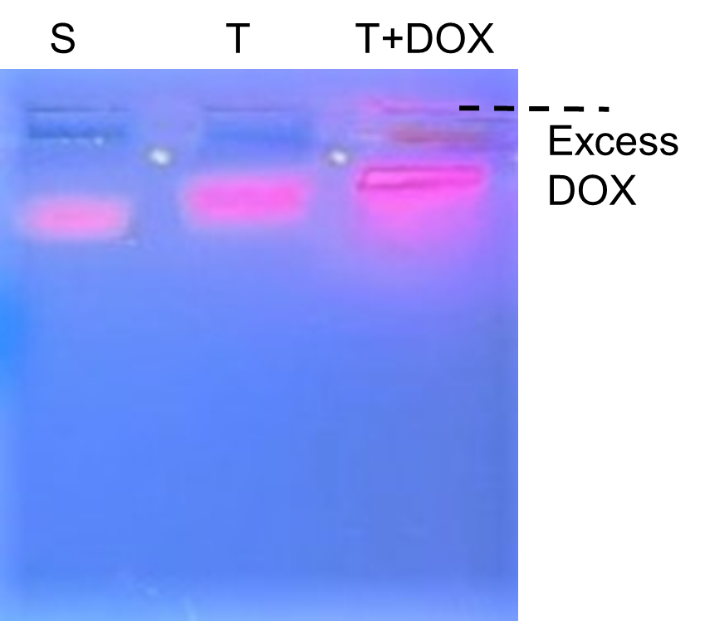
**

**Supplementary Figure S5.** Native 1.5 % agarose gel electrophoresis of M13 scaffold (lane S), targeted DNA origami (lane T), DOX loaded targeted DNA origami (lane T+DOX). The result confirms the binding of DOX with DNA origami structures.


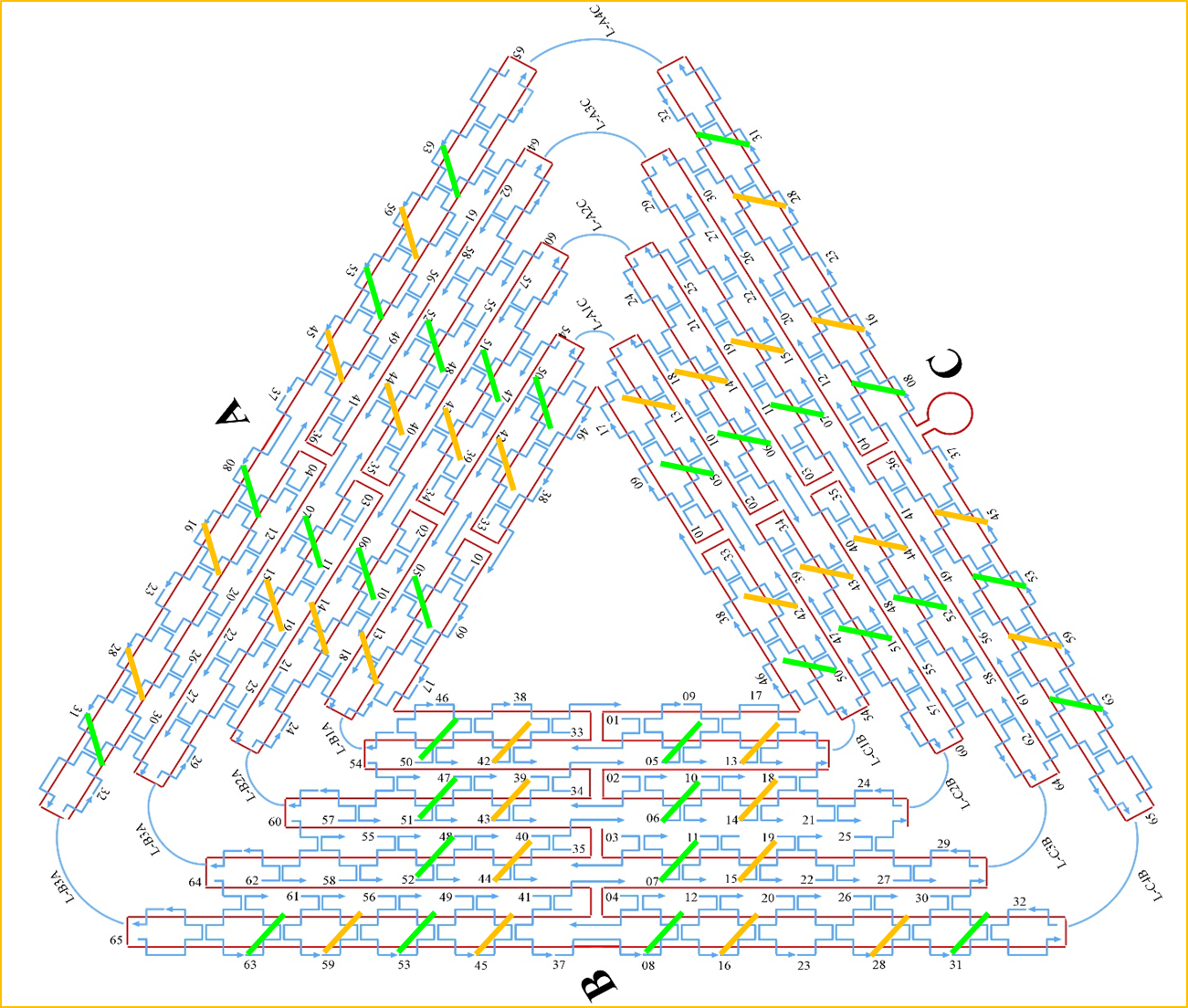


**Supplementary Figure S6.** Schematic representation of modified DNA origami. Green single stranded overhangs are complimentary to folate functionalized strands and orange single stranded overhangs are complimentary to NIR dye functionalized strands.

**DNA Origami staple strands and capture strands:**

Amine modified strand for Folate functionalization: CATGCCATACGGTCATTT-NH_2_

Amine modified strand for DyLight™ 690 (NIR Dye) functionalization: GAATGCGAATCAAGCTTT-NH_2_

A01, CGGGGTTTCCTCAAGAGAAGGATTTTGAATTA

A02, AGCGTCATGTCTCTGAATTTACCGACTACCTT

A03, TTCATAATCCCCTTATTAGCGTTTTTCTTACC,

A04, ATGGTTTATGTCACAATCAATAGATATTAAAC,

A05,TGACCGTATGGCATGTTTGATGATTAAGAGGCTGAGACTTGCTCAGTACCAGGCG,

A06, TGACCGTATGGCATGCCGGAACCCAGAATGGAAAGCGCAACATGGCT,

A07, TGACCGTATGGCATGAAAGACAACATTTTCGGTCATAGCCAAAATCA,

A08, TGACCGTATGGCATGGACGGGAGAATTAACTCGGAATAAGTTTATTTCCAGCGCC,

A09, GATAAGTGCCGTCGAGCTGAAACATGAAAGTATACAGGAG,

A10, TGTACTGGAAATCCTCATTAAAGCAGAGCCAC,

A11, CACCGGAAAGCGCGTTTTCATCGGAAGGGCGA,

A12, CATTCAACAAACGCAAAGACACCAGAACACCCTGAACAAA,

A13, GCTTGATTCGCATTCTTTAACGGTTCGGAACCTATTATTAGGGTTGATATAAGTA,

A14, GCTTGATTCGCATTCCTCAGAGCATATTCACAAACAAATTAATAAGT,

A15, GCTTGATTCGCATTCGGAGGGAATTTAGCGTCAGACTGTCCGCCTCC,

A16, GCTTGATTCGCATTCGTCAGAGGGTAATTGATGGCAACATATAAAAGCGATTGAG,

A17, TAGCCCGGAATAGGTGAATGCCCCCTGCCTATGGTCAGTG,

A18, CCTTGAGTCAGACGATTGGCCTTGCGCCACCC,

A19, TCAGAACCCAGAATCAAGTTTGCCGGTAAATA,

A20, TTGACGGAAATACATACATAAAGGGCGCTAATATCAGAGA,

A21, CAGAGCCAGGAGGTTGAGGCAGGTAACAGTGCCCG,

A22, ATTAAAGGCCGTAATCAGTAGCGAGCCACCCT,

A23, GATAACCCACAAGAATGTTAGCAAACGTAGAAAATTATTC,

A24, GCCGCCAGCATTGACACCACCCTC,

A25, AGAGCCGCACCATCGATAGCAGCATGAATTAT,

A26, CACCGTCACCTTATTACGCAGTATTGAGTTAAGCCCAATA,

A27, AGCCATTTAAACGTCACCAATGAACACCAGAACCA,

A28, GCTTGATTCGCATTCATAAGAGCAAGAAACATGGCATGATTAAGACTCCGACTTG,

A29, CCATTAGCAAGGCCGGGGGAATTA,

A30, GAGCCAGCGAATACCCAAAAGAACATGAAATAGCAATAGC,

A31, TGACCGTATGGCATGTATCTTACCGAAGCCCAAACGCAATAATAACGAAAATCACCAG,

A32, CAGAAGGAAACCGAGGTTTTTAAGAAAAGTAAGCAGATAGCCG,

A33, CCTTTTTTCATTTAACAATTTCATAGGATTAG,

A34, TTTAACCTATCATAGGTCTGAGAGTTCCAGTA,

A35, AGTATAAAATATGCGTTATACAAAGCCATCTT,

A36, CAAGTACCTCATTCCAAGAACGGGAAATTCAT,

A37, AGAGAATAACATAAAAACAGGGAAGCGCATTA,

A38, AAAACAAAATTAATTAAATGGAAACAGTACATTAGTGAAT,

A39, TTATCAAACCGGCTTAGGTTGGGTAAGCCTGT,

A40, TTAGTATCGCCAACGCTCAACAGTCGGCTGTC,

A41, TTTCCTTAGCACTCATCGAGAACAATAGCAGCCTTTACAG,

A42, GCTTGATTCGCATTCAGAGTCAAAAATCAATATATGTGATGAAACAAACATCAAG,

A43, GCTTGATTCGCATTCACTAGAAATATATAACTATATGTACGCTGAGA,

A44, GCTTGATTCGCATTCTCAATAATAGGGCTTAATTGAGAATCATAATT,

A45, GCTTGATTCGCATTCAACGTCAAAAATGAAAAGCAAGCCGTTTTTATGAAACCAA,

A46, GAGCAAAAGAAGATGAGTGAATAACCTTGCTTATAGCTTA,

A47, GATTAAGAAATGCTGATGCAAATCAGAATAAA,

A48, CACCGGAATCGCCATATTTAACAAAATTTACG,

A49, AGCATGTATTTCATCGTAGGAATCAAACGATTTTTTGTTT,

A50, TGACCGTATGGCATGACATAGCGCTGTAAATCGTCGCTATTCATTTCAATTACCT,

A51, TGACCGTATGGCATGGTTAAATACAATCGCAAGACAAAGCCTTGAAA,

A52, TGACCGTATGGCATGCCCATCCTCGCCAACATGTAATTTAATAAGGC,

A53, TGACCGTATGGCATGTCCCAATCCAAATAAGATTACCGCGCCCAATAAATAATAT,

A54, TCCCTTAGAATAACGCGAGAAAACTTTTACCGACC,

A55, GTGTGATAAGGCAGAGGCATTTTCAGTCCTGA,

A56, ACAAGAAAGCAAGCAAATCAGATAACAGCCATATTATTTA,

A57, GTTTGAAATTCAAATATATTTTAG,

A58, AATAGATAGAGCCAGTAATAAGAGATTTAATG,

A59, GCTTGATTCGCATTCGCCAGTTACAAAATAATAGAAGGCTTATCCGGTTATCAAC,

A60, TTCTGACCTAAAATATAAAGTACCGACTGCAGAAC,

A61, GCGCCTGTTATTCTAAGAACGCGATTCCAGAGCCTAATTT,

A62, TCAGCTAAAAAAGGTAAAGTAATT,

A63, TGACCGTATGGCATGACGCTAACGAGCGTCTGGCGTTTTAGCGAACCCAACATGT,

A64, ACGACAATAAATCCCGACTTGCGGGAGATCCTGAATCTTACCA,

A65, TGCTATTTTGCACCCAGCTACAATTTTGTTTTGAAGCCTTAAA,

B01, TCATATGTGTAATCGTAAAACTAGTCATTTTC,

B02, GTGAGAAAATGTGTAGGTAAAGATACAACTTT,

B03, GGCATCAAATTTGGGGCGCGAGCTAGTTAAAG,

B04, TTCGAGCTAAGACTTCAAATATCGGGAACGAG,

B05, TGACCGTATGGCATGACAGTCAAAGAGAATCGATGAACGACCCCGGTTGATAATC,

B06, TGACCGTATGGCATGATAGTAGTATGCAATGCCTGAGTAGGCCGGAG,

B07, TGACCGTATGGCATGAACCAGACGTTTAGCTATATTTTCTTCTACTA,

B08, TGACCGTATGGCATGGAATACCACATTCAACTTAAGAGGAAGCCCGATCAAAGCG,

B09, AGAAAAGCCCCAAAAAGAGTCTGGAGCAAACAATCACCAT,

B10, CAATATGACCCTCATATATTTTAAAGCATTAA,

B11, CATCCAATAAATGGTCAATAACCTCGGAAGCA,

B12, AACTCCAAGATTGCATCAAAAAGATAATGCAGATACATAA,

B13, GCTTGATTCGCATTCCGTTCTAGTCAGGTCATTGCCTGACAGGAAGATTGTATAA,

B14, GCTTGATTCGCATTCCAGGCAAGATAAAAATTTTTAGAATATTCAAC,

B15, GCTTGATTCGCATTCGATTAGAGATTAGATACATTTCGCAAATCATA,

B16, GCTTGATTCGCATTCCGCCAAAAGGAATTACAGTCAGAAGCAAAGCGCAGGTCAG,

B17, GCAAATATTTAAATTGAGATCTACAAAGGCTACTGATAAA,

B18, TTAATGCCTTATTTCAACGCAAGGGCAAAGAA,

B19, TTAGCAAATAGATTTAGTTTGACCAGTACCTT,

B20, TAATTGCTTTACCCTGACTATTATGAGGCATAGTAAGAGC,

B21, ATAAAGCCTTTGCGGGAGAAGCCTGGAGAGGGTAG,

B22, TAAGAGGTCAATTCTGCGAACGAGATTAAGCA,

B23, AACACTATCATAACCCATCAAAAATCAGGTCTCCTTTTGA,

B24, ATGACCCTGTAATACTTCAGAGCA,

B25, TAAAGCTATATAACAGTTGATTCCCATTTTTG,

B26, CGGATGGCACGAGAATGACCATAATCGTTTACCAGACGAC,

B27, TAATTGCTTGGAAGTTTCATTCCAAATCGGTTGTA,

B28, GCTTGATTCGCATTCGATAAAAACCAAAATATTAAACAGTTCAGAAATTAGAGCT,

B29, ACTAAAGTACGGTGTCGAATATAA,

B30, TGCTGTAGATCCCCCTCAAATGCTGCGAGAGGCTTTTGCA,

B31, TGACCGTATGGCATG AAAGAAGTTTTGCCAGCATAAATATTCATTGACTCAACATGTT,

B32, AATACTGCGGAATCGTAGGGGGTAATAGTAAAATGTTTAGACT,

B33, AGGGATAGCTCAGAGCCACCACCCCATGTCAA,

B34, CAACAGTTTATGGGATTTTGCTAATCAAAAGG,

B35, GCCGCTTTGCTGAGGCTTGCAGGGGAAAAGGT,

B36, GCGCAGACTCCATGTTACTTAGCCCGTTTTAA,

B37, ACAGGTAGAAAGATTCATCAGTTGAGATTTAG,

B38, CCTCAGAACCGCCACCCAAGCCCAATAGGAACGTAAATGA,

B39, ATTTTCTGTCAGCGGAGTGAGAATACCGATAT,

B40, ATTCGGTCTGCGGGATCGTCACCCGAAATCCG,

B41, CGACCTGCGGTCAATCATAAGGGAACGGAACAACATTATT,

B42, GCTTGATTCGCATTCAGACGTTACCATGTACCGTAACACCCCTCAGAACCGCCAC,

B43, GCTTGATTCGCATTCCACGCATAAGAAAGGAACAACTAAGTCTTTCC,

B44, GCTTGATTCGCATTCATTGTGTCTCAGCAGCGAAAGACACCATCGCC,

B45, GCTTGATTCGCATTCTTAATAAAACGAACTAACCGAACTGACCAACTCCTGATAA,

B46, AGGTTTAGTACCGCCATGAGTTTCGTCACCAGGATCTAAA,

B47, GTTTTGTCAGGAATTGCGAATAATCCGACAAT,

B48, GACAACAAGCATCGGAACGAGGGTGAGATTTG,

B49, TATCATCGTTGAAAGAGGACAGATGGAAGAAAAATCTACG,

B50, TGACCGTATGGCATGAGCGTAACTACAAACTACAACGCCTATCACCGTACTCAGG,

B51, TGACCGTATGGCATGTAGTTGCGAATTTTTTCACGTTGATCATAGTT,

B52, TGACCGTATGGCATGGTACAACGAGCAACGGCTACAGAGGATACCGA,

B53, TGACCGTATGGCATGACCAGTCAGGACGTTGGAACGGTGTACAGACCGAAACAAA,

B54, ACAGACAGCCCAAATCTCCAAAAAAAAATTTCTTA,

B55, AACAGCTTGCTTTGAGGACTAAAGCGATTATA,

B56, CCAAGCGCAGGCGCATAGGCTGGCAGAACTGGCTCATTAT,

B57, CGAGGTGAGGCTCCAAAAGGAGCC,

B58, ACCCCCAGACTTTTTCATGAGGAACTTGCTTT,

B59, GCTTGATTCGCATTCACCTTATGCGATTTTATGACCTTCATCAAGAGCATCTTTG,

B60, CGGTTTATCAGGTTTCCATTAAACGGGAATACACT,

B61, AAAACACTTAATCTTGACAAGAACTTAATCATTGTGAATT,

B62, GGCAAAAGTAAAATACGTAATGCC,

B63, TGACCGTATGGCATGTGGT TTAA TTTCAACTCGGATATTCATTACCCACGAAAGA,

B64, ACCAACCTAAAAAATCAACGTAACAAATAAATTGGGCTTGAGA,

B65, CCTGACGAGAAACACCAGAACGAGTAGGCTGCTCATTCAGTGA,

Link-A1C, TTAATTAATTTTTTACCATATCAAA,

Link-A2C, TTAATTTCATCTTAGACTTTACAA,

Link-A3C, CTGTCCAGACGTATACCGAACGA,

Link-A4C, TCAAGATTAGTGTAGCAATACT,

Link-B1A, TGTAGCATTCCTTTTATAAACAGTT,

Link-B2A, TTTAATTGTATTTCCACCAGAGCC,

Link-B3A, ACTACGAAGGCTTAGCACCATTA,

Link-B4A, ATAAGGCTTGCAACAAAGTTAC,

Link-C1B, GTGGGAACAAATTTCTATTTTTGAG,

Link-C2B, CGGTGCGGGCCTTCCAAAAACATT,

Link-C3B, ATGAGTGAGCTTTTAAATATGCA,

Link-C4B, ACTATTAAAGAGGATAGCGTCC,

Loop, GCGCTTAATGCGCCGCTACAGGGC,

C01, TCGGGAGATATACAGTAACAGTACAAATAATT,

C02, CCTGATTAAAGGAGCGGAATTATCTCGGCCTC,

C03, GCAAATCACCTCAATCAATATCTGCAGGTCGA,

C04, CGACCAGTACATTGGCAGATTCACCTGATTGC,

C05, TGACCGTATGGCATGTGGCAATTTTTAACGTCAGATGAAAACAATAACGGATTCG,

C06, TGACCGTATGGCATGAAGGAATTACAAAGAAACCACCAGTCAGATGA,

C07, TGACCGTATGGCATGGGACATTCACCTCAAATATCAAACACAGTTGA,

C08, TGACCGTATGGCATGTTGACGAGCACGTATACTGAAATGGATTATTTAATAAAAG,

C09, CCTGATTGCTTTGAATTGCGTAGATTTTCAGGCATCAATA,

C10, TAATCCTGATTATCATTTTGCGGAGAGGAAGG,

C11, TTATCTAAAGCATCACCTTGCTGATGGCCAAC,

C12, AGAGATAGTTTGACGCTCAATCGTACGTGCTTTCCTCGTT,

C13, GCTTGATTCGCATTCGATTATACACAGAAATAAAGAAATACCAAGTTACAAAATC,

C14, GCTTGATTCGCATTCTAGGAGCATAAAAGTTTGAGTAACATTGTTTG,

C15, GCTTGATTCGCATTCTGACCTGACAAATGAAAAATCTAAAATATCTT,

C16, GCTTGATTCGCATTCAGAATCAGAGCGGGAGATGGAAATACCTACATAACCCTTC,

C17, GCGCAGAGGCGAATTAATTATTTGCACGTAAATTCTGAAT,

C18, AATGGAAGCGAACGTTATTAATTTCTAACAAC,

C19, TAATAGATCGCTGAGAGCCAGCAGAAGCGTAA,

C20, GAATACGTAACAGGAAAAACGCTCCTAAACAGGAGGCCGA,

C21, TCAATAGATATTAAATCCTTTGCCGGTTAGAACCT,

C22, CAATATTTGCCTGCAACAGTGCCATAGAGCCG,

C23, TTAAAGGGATTTTAGATACCGCCAGCCATTGCGGCACAGA,

C24, ACAATTCGACAACTCGTAATACAT,

C25, TTGAGGATGGTCAGTATTAACACCTTGAATGG,

C26, CTATTAGTATATCCAGAACAATATCAGGAACGGTACGCCA,

C27, CGCGAACTAAAACAGAGGTGAGGCTTAGAAGTATT,

C28, GCTTGATTCGCATTCGAATCCTGAGAAGTGTATCGGCCTTGCTGGTACTTTAATG,

C29, ACCACCAGCAGAAGATGATAGCCC,

C30, TAAAACATTAGAAGAACTCAAACTTTTTATAATCAGTGAG,

C31, TGACCGTATGGCATGGCCACCGAGTAAAAGAACATCACTTGCCTGAGCGCCATTAAAA,

C32, TCTTTGATTAGTAATAGTCTGTCCATCACGCAAATTAACCGTT,

C33, CGCGTCTGATAGGAACGCCATCAACTTTTACA,

C34, AGGAAGATGGGGACGACGACAGTAATCATATT,

C35, CTCTAGAGCAAGCTTGCATGCCTGGTCAGTTG,

C36, CCTTCACCGTGAGACGGGCAACAGCAGTCACA,

C37, CGAGAAAGGAAGGGAAGCGTACTATGGTTGCT,

C38, GCTCATTTTTTAACCAGCCTTCCTGTAGCCAGGCATCTGC,

C39, CAGTTTGACGCACTCCAGCCAGCTAAACGACG,

C40, GCCAGTGCGATCCCCGGGTACCGAGTTTTTCT,

C41, TTTCACCAGCCTGGCCCTGAGAGAAAGCCGGCGAACGTGG,

C42, GCTTGATTCGCATTCGTAACCGTCTTTCATCAACATTAAAATTTTTGTTAAATCA,

C43, GCTTGATTCGCATTCACGTTGTATTCCGGCACCGCTTCTGGCGCATC,

C44, GCTTGATTCGCATTCCCAGGGTGGCTCGAATTCGTAATCCAGTCACG,

C45, GCTTGATTCGCATTCTAGAGCTTGACGGGGAGTTGCAGCAAGCGGTCATTGGGCG,

C46, GTTAAAATTCGCATTAATGTGAGCGAGTAACACACGTTGG,

C47, TGTAGATGGGTGCCGGAAACCAGGAACGCCAG,

C48, GGTTTTCCATGGTCATAGCTGTTTGAGAGGCG,

C49, GTTTGCGTCACGCTGGTTTGCCCCAAGGGAGCCCCCGATT,

C50, TGACCGTATGGCATGGGATAGGTACCCGTCGGATTCTCCTAAACGTTAATATTTT,

C51, TGACCGTATGGCATGAGTTGGGTCAAAGCGCCATTCGCCCCGTAATG,

C52, TGACCGTATGGCATGCGCGCGGGCCTGTGTGAAATTGTTGGCGATTA,

C53, TGACCGTATGGCATGCTAAATCGGAACCCTAAGCAGGCGAAAATCCTTCGGCCAA,

C54, CGGCGGATTGAATTCAGGCTGCGCAACGGGGGATG,

C55, TGCTGCAAATCCGCTCACAATTCCCAGCTGCA,

C56, TTAATGAAGTTTGATGGTGGTTCCGAGGTGCCGTAAAGCA,

C57, TGGCGAAATGTTGGGAAGGGCGAT,

C58, TGTCGTGCACACAACATACGAGCCACGCCAGC,

C59, GCTTGATTCGCATTCCAAGTTTTTTGGGGTCGAAATCGGCAAAATCCGGGAAACC,

C60, TCTTCGCTATTGGAAGCATAAAGTGTATGCCCGCT,

C61, TTCCAGTCCTTATAAATCAAAAGAGAACCATCACCCAAAT,

C62, GCGCTCACAAGCCTGGGGTGCCTA,

C63, TGACCGTATGGCATGCGATGGCCCACTACGTATAGCCCGAGATAGGGATTGCGTT,

C64, AACTCACATTATTGAGTGTTGTTCCAGAAACCGTCTATCAGGG,

C65, ACGTGGACTCCAACGTCAAAGGGCGAATTTGGAACAAGAGTCC,
